# A second monoclinic polymorph of 2-amino-4,6-dichloro­pyrimidine

**DOI:** 10.1107/S1600536808023714

**Published:** 2008-08-06

**Authors:** Hoong-Kun Fun, Suchada Chantrapromma, Subrata Jana, Rinku Chakrabarty, Shyamaprosad Goswami

**Affiliations:** a X-ray Crystallography Unit, School of Physics, Universiti Sains Malaysia, 11800 USM, Penang, Malaysia; b Crystal Materials Research Unit, Department of Chemistry, Faculty of Science, Prince of Songkla University, Hat-Yai, Songkhla 90112, Thailand; c Department of Chemistry, Bengal Engineering and Science University’, Shibpur, Howrah, India 711 103

## Abstract

The title chloro-substituted 2-amino­pyrimidine, C_4_H_3_Cl_2_N_3_, is a second monoclinic polymorph of this compound which crystallizes in the space group *C*2/*c*. The structure was previously reported [Clews & Cochran (1948 ▶). *Acta Cryst*. **1**, 4–11] in the space group *P*21/*a*. There are two crystallographically independent mol­ecules in the asymmetric unit and each mol­ecule is planar. The dihedral angle between the two pyrimidine rings is 30.71 (12)°. In the crystal structure, mol­ecules are linked *via* N—H⋯N inter­molecular hydrogen bonds, forming infinite one-dimensional chains along the *a* axis. These hydrogen bonds generate *R* ^2^_2_(8) ring motifs. The chains are stacked along the *b* axis.

## Related literature

For bond-length data, see: Allen *et al.* (1987 ▶). For details of hydrogen-bond motifs, see: Bernstein *et al.* (1995 ▶). For related structures, see: the polymorph reported by Clews & Cochran (1948 ▶); Low *et al.* (2002 ▶). For applications of pyrimidine compounds and their supra­molecular chemistry, see, for example: Blackburn & Gait (1996 ▶); Brown (1988 ▶); Hurst (1980 ▶); Goswami *et al.* (2008*a* ▶,*b* ▶); Ligthart *et al.* (2005 ▶); Sherrington & Taskinen (2001 ▶).
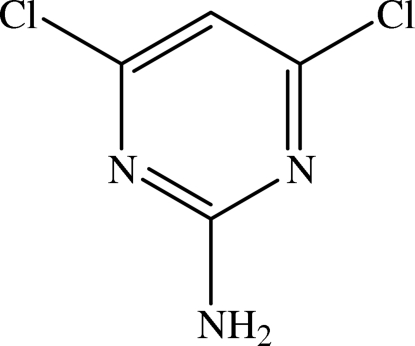


## Experimental

### 

#### Crystal data

C_4_H_3_Cl_2_N_3_
*M* *_r_* = 163.99
Monoclinic, 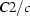

*a* = 32.060 (4) Å
*b* = 3.8045 (6) Å
*c* = 21.302 (3) Å
β = 102.193 (7)°
*V* = 2539.6 (6) Å^3^
*Z* = 16
Mo *K*α radiation
μ = 0.92 mm^−1^
*T* = 296 (2) K
0.57 × 0.14 × 0.02 mm

#### Data collection

Bruker SMART APEX2 CCD area-detector diffractometer
Absorption correction: multi-scan (*SADABS*; Bruker, 2005 ▶) *T*_min_ = 0.620, *T*_max_ = 0.985
12772 measured reflections
2886 independent reflections
1875 reflections with *I* > 2σ(*I*)
*R*_int_ = 0.051

#### Refinement

*R*[*F* ^2^ > 2σ(*F* ^2^)] = 0.040
*wR*(*F* ^2^) = 0.098
*S* = 1.02
2886 reflections
187 parameters
All H-atom parameters refined
Δρ_max_ = 0.22 e Å^−3^
Δρ_min_ = −0.24 e Å^−3^

### 

Data collection: *APEX2* (Bruker, 2005 ▶); cell refinement: *APEX2*; data reduction: *SAINT* (Bruker, 2005 ▶); program(s) used to solve structure: *SHELXTL* (Sheldrick, 2008 ▶); program(s) used to refine structure: *SHELXTL*; molecular graphics: *SHELXTL*; software used to prepare material for publication: *SHELXTL* and *PLATON* (Spek, 2003 ▶).

## Supplementary Material

Crystal structure: contains datablocks global, I. DOI: 10.1107/S1600536808023714/sj2524sup1.cif

Structure factors: contains datablocks I. DOI: 10.1107/S1600536808023714/sj2524Isup2.hkl

Additional supplementary materials:  crystallographic information; 3D view; checkCIF report

## Figures and Tables

**Table 1:** Hydrogen-bond geometry (Å, °)

*D*—H⋯*A*	*D*—H	H⋯*A*	*D*⋯*A*	*D*—H⋯*A*
N3*A*—H2*NA*⋯N1*A*^i^	0.75 (3)	2.43 (3)	3.172 (3)	176 (2)
N3*A*—H1*NA*⋯N2*B*^i^	0.87 (3)	2.33 (3)	3.201 (3)	172 (2)
N3*B*—H1*NB*⋯N2*A*^i^	0.87 (3)	2.39 (3)	3.253 (4)	174 (3)
N3*B*—H2*NB*⋯N1*B*^ii^	0.84 (3)	2.41 (3)	3.242 (3)	172 (3)

Symmetry codes: (i) 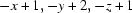; (ii) 
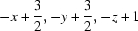
.

**Fig. 1.**
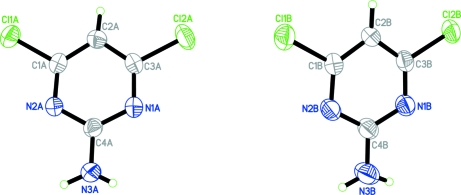
The molecular structure of the title compound, showing 50% probability displacement ellipsoids and the atomic numbering.

**Fig. 2.**
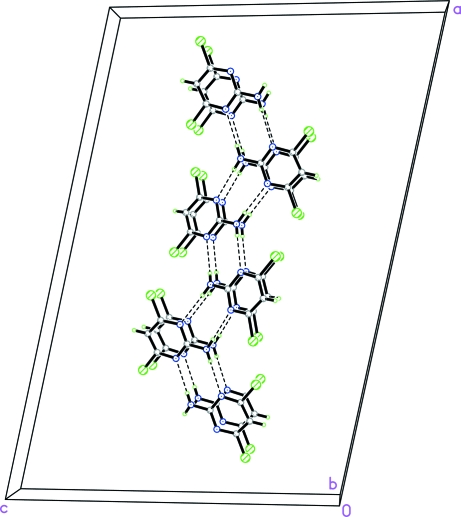
The crystal packing of (I), viewed approximately along the b axis showing one-dimensional chains along the a axis. Hydrogen bonds were shown as dashed lines.

## supplementary crystallographic information

### Comment

Functionalized pyrimidines play a major role in the synthesis of different drug molecules and of naturally occurring pyrimidine bases (Blackburn & Gait, 1996; Brown, 1988; Hurst, 1980). Substituted pyrimidines are also very important for studies on multiple hydrogen bonding interactions in molecular recognition and supramolecular chemistry (Sherrington & Taskinen, 2001; Goswami *et al.*, 2008*a*,*b*; Ligthart *et al.*, 20050). In this work we report the crystal structure of the title compound, Fig 1,  which is a second monoclinic polymorph of 2-amino-4,6-dichloropyrimidine.

The crystal structure of the title compound (I) was previously reported by Clews & Cochran (1948) in the monoclinic space group *P*21/a, with a = 16.447, b = 3.845, c = 10.283 Å, β = 107.58° and *Z* = 4. In the present work, the compound crystallized out in the monoclinic space group *C*2/c with *Z* = 16. There are two crystallographically independent molecules in the asymmetric unit, *A* and *B*, (see Fig. 1) with slightly different bond lengths and bond angles. Both molecules *A* and *B* are planar with maximum deviations of 0.005 (2) Å for atom N2A in *A* and 0.009 (2) Å for atom C2B in *B*. The dihedral angle between the two pyrimidine rings is 30.71 (12)°. The amino group acts as a double donor in N—H···N hydrogen bonds, while the two ring N atoms (N1 and N2) act as the acceptors. The molecules are linked *via* N—H···N intermolecular hydrogen bonds to form infinite one-dimensional chains along the *a* axis, Table 1. These hydrogen bonds generate *R*^2^_2_(8) ring motifs (Bernstein *at al*., 1995) (Fig. 2). Interestingly, the Cl atoms do not form N—H···Cl hydrogen bonds. The closest Cl···Cl distance is 3.3635 (11) Å [3.37 Å in Clews & Cochran (1948)]. The bond lengths and angles in (I) are within normal ranges (Allen *et al.*, 1987) and comparable to those found in related structures (Clews & Cochran, 1948; Low *et al.*, 2002).

In the crystal packing shown in Fig. 2, the [1 0 0] molecular chains are stacked along the *b* axis.

### Experimental

Phosphorus oxy-chloride (POCl_3_) (25 ml) was added to anhydrous 2-amino-4,6-dioxopyrimidine (6 g) and the mixture refluxed at 383 K for 12 h. Excess POCl_3_ was distilled off. The solid residue was neutralized using KOH solution in an ice bath and saturated NaHCO_3_ solution was added. The solid residue was filtered off, extracted with CHCl_3_ and the solution was dried over Na_2_SO_4_ and then concentrated under vacuum. The crude product was purified by column chromatography using 20% ethyl acetate in petroleum ether as eluent and the title compound (I) (4.29 g, 61%) was isolated. Single crystals were grown by slow evaporation of a CH_2_Cl_2_/ethanol (*v*/*v* 3:1) solution, Mp. 492–494 K.

### Refinement

All H atoms were located in a difference map and freely refined isotropically. The highest residual electron density peak is located at 1.00 Å from N2A and the deepest hole is located at 0.81 Å from H2NA.

### Crystal data

**Table:**

C_4_H_3_Cl_2_N_3_	*F*_000_ = 1312
*M**_r_* = 163.99	*D*_x_ = 1.716 Mg m^−^^3^
Monoclinic, *C*2/*c*	Melting point = 492–494 K
Hall symbol: -C 2yc	Mo *K*α radiation λ = 0.71073 Å
*a* = 32.060 (4) Å	Cell parameters from 2886 reflections
*b* = 3.8045 (6) Å	θ = 1.3–27.5º
*c* = 21.302 (3) Å	µ = 0.92 mm^−^^1^
β = 102.193 (7)º	*T* = 296 (2) K
*V* = 2539.6 (6) Å^3^	Block, colorless
*Z* = 16	0.57 × 0.14 × 0.02 mm

### Data collection

**Table:**

Bruker SMART APEX2 CCD area-detector diffractometer	2886 independent reflections
Radiation source: fine-focus sealed tube	1875 reflections with *I* > 2σ(*I*)
Monochromator: graphite	*R*_int_ = 0.051
Detector resolution: 8.33 pixels mm^-1^	θ_max_ = 27.5º
*T* = 296(2) K	θ_min_ = 1.3º
ω scans	*h* = −40→40
Absorption correction: multi-scan(SADABS; Bruker, 2005)	*k* = −4→4
*T*_min_ = 0.620, *T*_max_ = 0.985	*l* = −27→27
12772 measured reflections	

### Refinement

**Table:**

Refinement on *F*^2^	Secondary atom site location: difference Fourier map
Least-squares matrix: full	Hydrogen site location: inferred from neighbouring sites
*R*[*F*^2^ > 2σ(*F*^2^)] = 0.040	All H-atom parameters refined
*wR*(*F*^2^) = 0.098	*w* = 1/[σ^2^(*F*_o_^2^) + (0.0403*P*)^2^] where *P* = (*F*_o_^2^ + 2*F*_c_^2^)/3
*S* = 1.02	(Δ/σ)_max_ = 0.001
2886 reflections	Δρ_max_ = 0.22 e Å^−^^3^
187 parameters	Δρ_min_ = −0.24 e Å^−^^3^
Primary atom site location: structure-invariant direct methods	Extinction correction: none

### Special details

**Table:**

Geometry. All e.s.d.'s (except the e.s.d. in the dihedral angle between two l.s. planes) are estimated using the full covariance matrix. The cell e.s.d.'s are taken into account individually in the estimation of e.s.d.'s in distances, angles and torsion angles; correlations between e.s.d.'s in cell parameters are only used when they are defined by crystal symmetry. An approximate (isotropic) treatment of cell e.s.d.'s is used for estimating e.s.d.'s involving l.s. planes.
Refinement. Refinement of *F*^2^ against ALL reflections. The weighted *R*-factor *wR* and goodness of fit *S* are based on *F*^2^, conventional *R*-factors *R* are based on *F*, with *F* set to zero for negative *F*^2^. The threshold expression of *F*^2^ > σ(*F*^2^) is used only for calculating *R*-factors(gt) *etc*. and is not relevant to the choice of reflections for refinement. *R*-factors based on *F*^2^ are statistically about twice as large as those based on *F*, and *R*- factors based on ALL data will be even larger.

### Fractional atomic coordinates and isotropic or equivalent isotropic displacement parameters (Å^2^)

**Table:**

	*x*	*y*	*z*	*U*_iso_*/*U*_eq_	
Cl1A	0.330814 (19)	0.45738 (18)	0.35093 (3)	0.0475 (2)	
Cl2A	0.49584 (2)	0.5271 (2)	0.33992 (4)	0.0572 (2)	
N1A	0.46421 (6)	0.7708 (5)	0.43348 (9)	0.0371 (5)	
N2A	0.38986 (6)	0.7401 (5)	0.43849 (9)	0.0349 (5)	
N3A	0.43941 (8)	0.9927 (7)	0.51907 (11)	0.0490 (6)	
H2NA	0.4619 (8)	1.056 (7)	0.5293 (12)	0.033 (8)*	
H1NA	0.4174 (9)	1.026 (7)	0.5366 (14)	0.057 (9)*	
C1A	0.38306 (7)	0.5786 (6)	0.38271 (11)	0.0338 (6)	
C2A	0.41389 (8)	0.5008 (7)	0.34867 (12)	0.0377 (6)	
H2A	0.4102 (7)	0.386 (6)	0.3120 (11)	0.034 (7)*	
C3A	0.45423 (7)	0.6088 (7)	0.37766 (11)	0.0353 (6)	
C4A	0.43110 (7)	0.8296 (7)	0.46279 (11)	0.0350 (6)	
Cl1B	0.58263 (2)	1.02542 (19)	0.31034 (3)	0.0510 (2)	
Cl2B	0.73894 (2)	0.50031 (18)	0.32094 (3)	0.0474 (2)	
N1B	0.71199 (6)	0.7404 (6)	0.41918 (9)	0.0389 (5)	
N2B	0.64127 (6)	0.9690 (6)	0.41463 (9)	0.0406 (5)	
N3B	0.69119 (9)	0.9534 (8)	0.50908 (11)	0.0589 (7)	
H1NB	0.6706 (10)	1.049 (8)	0.5237 (16)	0.080 (12)*	
H2NB	0.7156 (10)	0.881 (9)	0.5264 (16)	0.075 (11)*	
C1B	0.63348 (7)	0.9103 (6)	0.35235 (12)	0.0364 (6)	
C2B	0.66172 (7)	0.7709 (7)	0.31888 (11)	0.0374 (6)	
H2B	0.6550 (7)	0.753 (7)	0.2743 (12)	0.047 (7)*	
C3B	0.70066 (7)	0.6887 (7)	0.35702 (11)	0.0361 (6)	
C4B	0.68134 (8)	0.8851 (7)	0.44634 (11)	0.0403 (6)	

### Atomic displacement parameters (Å^2^)

**Table:**

	*U*^11^	*U*^22^	*U*^33^	*U*^12^	*U*^13^	*U*^23^
Cl1A	0.0320 (3)	0.0586 (5)	0.0491 (4)	−0.0065 (3)	0.0027 (3)	−0.0029 (3)
Cl2A	0.0442 (4)	0.0698 (5)	0.0658 (5)	−0.0003 (4)	0.0298 (3)	−0.0132 (4)
N1A	0.0293 (10)	0.0422 (13)	0.0402 (11)	0.0003 (10)	0.0086 (9)	−0.0014 (11)
N2A	0.0295 (10)	0.0416 (13)	0.0339 (10)	0.0028 (10)	0.0073 (8)	−0.0013 (10)
N3A	0.0351 (14)	0.0707 (19)	0.0418 (13)	−0.0073 (14)	0.0093 (11)	−0.0161 (13)
C1A	0.0293 (12)	0.0320 (14)	0.0385 (13)	−0.0009 (11)	0.0040 (10)	0.0039 (11)
C2A	0.0378 (14)	0.0402 (16)	0.0355 (13)	−0.0012 (13)	0.0087 (11)	−0.0057 (13)
C3A	0.0338 (13)	0.0354 (14)	0.0396 (13)	0.0033 (12)	0.0141 (10)	0.0001 (12)
C4A	0.0327 (13)	0.0404 (15)	0.0315 (12)	−0.0020 (12)	0.0060 (10)	0.0013 (12)
Cl1B	0.0344 (4)	0.0669 (5)	0.0514 (4)	0.0072 (4)	0.0083 (3)	0.0056 (4)
Cl2B	0.0358 (3)	0.0566 (4)	0.0546 (4)	−0.0001 (3)	0.0204 (3)	−0.0103 (3)
N1B	0.0355 (11)	0.0438 (13)	0.0386 (11)	0.0036 (11)	0.0109 (9)	0.0016 (10)
N2B	0.0388 (12)	0.0481 (14)	0.0377 (11)	0.0044 (11)	0.0143 (9)	0.0001 (11)
N3B	0.0534 (17)	0.087 (2)	0.0368 (13)	0.0187 (16)	0.0102 (12)	−0.0034 (13)
C1B	0.0315 (13)	0.0386 (15)	0.0407 (13)	0.0001 (12)	0.0112 (10)	0.0014 (12)
C2B	0.0352 (14)	0.0465 (17)	0.0328 (13)	−0.0025 (13)	0.0123 (11)	−0.0036 (13)
C3B	0.0329 (13)	0.0369 (15)	0.0416 (14)	−0.0031 (12)	0.0151 (11)	−0.0009 (12)
C4B	0.0398 (15)	0.0477 (16)	0.0352 (13)	0.0043 (13)	0.0122 (11)	0.0023 (12)

### Geometric parameters (Å, °)

**Table:**

Cl1A—C1A	1.731 (2)	Cl1B—C1B	1.742 (2)
Cl2A—C3A	1.725 (2)	Cl2B—C3B	1.735 (2)
N1A—C3A	1.317 (3)	N1B—C3B	1.312 (3)
N1A—C4A	1.359 (3)	N1B—C4B	1.358 (3)
N2A—C1A	1.314 (3)	N2B—C1B	1.316 (3)
N2A—C4A	1.357 (3)	N2B—C4B	1.357 (3)
N3A—C4A	1.326 (3)	N3B—C4B	1.332 (3)
N3A—H2NA	0.75 (2)	N3B—H1NB	0.87 (3)
N3A—H1NA	0.87 (3)	N3B—H2NB	0.84 (3)
C1A—C2A	1.376 (3)	C1B—C2B	1.372 (3)
C2A—C3A	1.373 (3)	C2B—C3B	1.374 (3)
C2A—H2A	0.88 (2)	C2B—H2B	0.93 (2)
			
C3A—N1A—C4A	115.3 (2)	C3B—N1B—C4B	114.8 (2)
C1A—N2A—C4A	115.11 (19)	C1B—N2B—C4B	114.9 (2)
C4A—N3A—H2NA	114 (2)	C4B—N3B—H1NB	114 (2)
C4A—N3A—H1NA	115.4 (19)	C4B—N3B—H2NB	112 (2)
H2NA—N3A—H1NA	130 (3)	H1NB—N3B—H2NB	134 (3)
N2A—C1A—C2A	125.2 (2)	N2B—C1B—C2B	125.7 (2)
N2A—C1A—Cl1A	116.12 (18)	N2B—C1B—Cl1B	115.67 (18)
C2A—C1A—Cl1A	118.68 (19)	C2B—C1B—Cl1B	118.62 (19)
C3A—C2A—C1A	114.3 (2)	C1B—C2B—C3B	113.4 (2)
C3A—C2A—H2A	118.9 (14)	C1B—C2B—H2B	121.6 (15)
C1A—C2A—H2A	126.7 (15)	C3B—C2B—H2B	124.8 (14)
N1A—C3A—C2A	124.9 (2)	N1B—C3B—C2B	125.9 (2)
N1A—C3A—Cl2A	116.14 (18)	N1B—C3B—Cl2B	115.99 (17)
C2A—C3A—Cl2A	118.95 (19)	C2B—C3B—Cl2B	118.12 (18)
N3A—C4A—N2A	117.1 (2)	N3B—C4B—N2B	116.9 (2)
N3A—C4A—N1A	117.7 (2)	N3B—C4B—N1B	117.8 (2)
N2A—C4A—N1A	125.2 (2)	N2B—C4B—N1B	125.3 (2)

### Hydrogen-bond geometry (Å, °)

**Table:**

*D*—H···*A*	*D*—H	H···*A*	*D*···*A*	*D*—H···*A*
N3A—H2NA···N1A^i^	0.75 (3)	2.43 (3)	3.172 (3)	176 (2)
N3A—H1NA···N2B^i^	0.87 (3)	2.33 (3)	3.201 (3)	172 (2)
N3B—H1NB···N2A^i^	0.87 (3)	2.39 (3)	3.253 (4)	174 (3)
N3B—H2NB···N1B^ii^	0.84 (3)	2.41 (3)	3.242 (3)	172 (3)

Symmetry codes: (i) −*x*+1, −*y*+2, −*z*+1; (ii) −*x*+3/2, −*y*+3/2, −*z*+1.
